# Strategies for Recruiting Migrants to Participate in a Sexual Health Survey: Methods, Results, and Lessons

**DOI:** 10.3390/ijerph191912213

**Published:** 2022-09-26

**Authors:** Daniel Vujcich, Graham Brown, Jo Durham, Zhihong Gu, Lisa Hartley, Roanna Lobo, Limin Mao, Piergiorgio Moro, Vivienne Pillay, Amy B. Mullens, Enaam Oudih, Meagan Roberts, Caitlin Wilshin, Alison Reid

**Affiliations:** 1 School of Population Health, Curtin University, Bentley, WA 6102, Australia; 2 Centre for Social Impact, UNSW, Sydney, NSW 2052, Australia; 3 Centre for Healthcare Transformation, Australian Centre for Health Services Innovation, School of Public Health and Social Work, Queensland University of Technology, Kelvin Grove, QLD 4059, Australia; 4 Ethnic Communities Council of Queensland, West End, QLD 4101, Australia; 5 Centre for Human Rights Education, Curtin University, Bentley, WA 6102, Australia; 6 Centre for Social Research in Health, UNSW, Sydney, NSW 2052, Australia; 7 Multicultural Health Support Service, Centre for Culture, Ethnicity and Health, Richmond, VIC 3121, Australia; 8 Ethnic Communities Council of WA, North Perth, WA 6006, Australia; 9 Centre for Health Research, School of Psychology & Wellbeing, University of Southern Queensland, Ipswich, QLD 4305, Australia; 10 Relationships Australia South Australia, Hindmarsh, SA 5007, Australia

**Keywords:** migrants, ethnic groups, surveys and questionnaires, health surveys, sexual health, research design, recruitment, community participation, social media

## Abstract

In this article, we describe the approaches taken to recruit adult migrants living in Australia for a sexual health and blood-borne virus survey (paper and online) and present data detailing the outcomes of these approaches. The purpose was to offer guidance to redress the under-representation of migrants in public health research. Methods of recruitment included directly contacting people in individual/organizational networks, social media posts/advertising, promotion on websites, and face-to-face recruitment at public events/venues. Search query strings were used to provide information about an online referral source, and project officers kept records of activities and outcomes. Descriptive statistical analyses were used to determine respondent demographic characteristics, proportions recruited to complete the paper and online surveys, and sources of referral. Logistic regression analyses were run to predict online participation according to demographic characteristics. The total sample comprised 1454 African and Asian migrants, with 59% identifying as female. Most respondents (72%) were recruited to complete the paper version of the survey. Face-to-face invitations resulted in the highest number of completions. Facebook advertising did not recruit large numbers of respondents. Same-sex attraction and age (40–49 years) were statistically significant predictors of online completion. We encourage more researchers to build the evidence base on ways to produce research that reflects the needs and perspectives of minority populations who often bear the greatest burden of disease.

## 1. Introduction

Migrant populations are underrepresented in health and medical research, despite the fact that they (1) comprise a growing population in high- and middle-income countries and (2) have been identified as a priority group on the basis that they experience comparatively lower levels of access to health services and poorer health outcomes [1,2]. The underrepresentation of migrants and other minority groups in research is scientifically and ethically problematic; it casts doubt on the generalizability of study findings, compromises our ability to monitor disparities, and impedes efforts to tailor governmental, clinical, and community interventions to achieve health equity for all [1,2,3]. 

Historically, the reasons given for the lack of representation of migrants and other minority groups tended to focus on the attitudes and characteristics of those groups. Numerous studies positioned underrepresented populations as ‘hard to reach’ on the basis that they were perceived to be mistrustful of researchers, fearful of authority, wary of stigmatization, or have low levels of literacy in general and health literacy in particular [4,5]. However, there is a growing shift away from defining the ‘problem’ in terms of the unwillingness of participants to engage in research. Instead, more attention is being given to the need for researchers to adapt their study designs and recruitment practices to be more inclusive of and relevant to a broader range of people and lived experiences [4,5,6,7,8,9]. 

A rich body of research is emerging that demonstrates that recruitment of migrants and other minority groups into scientific studies is possible if tailored strategies are adopted [6,7,8,9,10,11]. However, minority groups are neither homogenous nor static, and it cannot be assumed that recruitment strategies adopted in one study will necessarily be successful in another.

One literature review demonstrated that the experiences of recruiting South Asian participants in the United Kingdom was affected by intersectional factors, such as age, social class, and education level [10]. In addition to tailoring approaches to reflect these internal diversities, different study designs may also necessitate different approaches. For instance, recruitment strategies for clinical trials might vary from those used in other types of studies due to differences in potential risks and benefits to participants and the relationship between recruiter (often a physician) and participant (often a patient) [11]. The nature of the research topic may also necessitate further adaptations to recruitment, with studies noting how sensitive or stigmatizing subjects (e.g., tuberculosis among Somali migrants living in the United Kingdom) may require unique approaches [12]. Additionally, with respect to the use of gratuities in the recruitment phase, a systematic review noted that they can “have negative connotations in some cultures” (i.e., promote a feeling of being “bought”) but warned against generalizing across groups because different cultural factors may produce different reactions and outcomes [4,13]. Although it is common for multiple recruitment strategies to be combined in research involving migrant populations, it can be difficult to discern from the literature which specific strategies (or combinations thereof) were most effective [14].

In light of these complexities and the pressing need to redress the problem of under-representation in research, there have been calls for researchers to provide more detailed descriptions of the recruitment methods used and their outcomes [6,7]. More transparent and fulsome reporting of recruitment methods will provide guidance to future researchers, as well as build our knowledge about which recruitment methods work for whom and in which contexts. In building the evidence base in this way, the goal is to ensure that research can be conducted in a manner that is not only sufficiently inclusive of the populations being studied but also time- and resource-efficient and culturally appropriate. 

In this article, we describe the approach taken to recruit a sample of adult migrants living in Australia for a sexual health and blood-borne virus (SHBBV) survey and present data detailing the outcomes of these approaches. The study was not designed to experimentally compare the effectiveness of different strategies of recruitment; rather, our primary aims were to explore and rigorously document the feasibility of collecting data from a diverse population of migrants to measure SHBBV knowledge, attitudes, and practices in Australia; inform research design for future periodic data collection in this population; and contribute to building a credible evidence base. Specifically, the objectives of this article are to:

1. Ascertain whether it is possible to recruit at least 1100 African and Asian migrants living in Australia from a diverse range of countries of origin, age groups, and gender identities to participate in a self-administered paper and online SHBBV survey;
2. Document the outcomes, strengths, and challenges associated with recruitment through social media, face-to-face recruitment at public events and venues, and direct invitations to networks;
3. Determine the acceptability of providing gratuities to migrants during recruitment to an SHBBV survey study; and
4. Identify other barriers to both recruitment and research on recruitment and provide recommendations to assist future researchers in overcoming these barriers.

## 2. Materials and Methods

Recruitment to the survey commenced in early September 2020 and concluded at the end of May 2021, during which time the COVID-19 pandemic was occurring. The survey comprised 48 items and was offered in five languages (English, Khmer, Vietnamese, Traditional Chinese, and Simplified Chinese). Details of the steps taken to pretest and translate the survey instrument are documented elsewhere [15,16]. The survey could be self-administered in two formats: paper or online. Although an offline version of the survey was also made available for completion on communal iPads, the devices were not ultimately used due to concerns about COVID-19 transmission. 

The source population for the study comprised people older than the age of 18 years who were (a) living in Australia at the time of the study and (b) born in Sub-Saharan Africa (SSA), South-East Asia (SEA), or North-East Asia (NEA). Non-probability quota sampling was conducted with a view to ensuring that the sample was equally distributed by gender, birth region, and age group to facilitate group comparisons. 

The recruitment process was designed to be primarily driven by partner organizations who had experience working with migrant communities in Australia. These partner organizations (Relationships Australia; Ethnic Communities Council of Queensland; Ethnic Communities Council of Western Australia; and the Centre for Culture, Ethnicity and Health) were provided with funding to employ project officers or utilize existing staff to recruit people into the study. The project officers undertook training to familiarize themselves with the study and ethical obligations around data collection. 

Different recruitment strategies were adopted for different survey formats (online and print), as described below. There were also variations between strategies adopted in each participating state, as summarized in Table 1. For instance, funding was not available to enable partner organizations in New South Wales to employ project officers, and as such, recruitment in that state was online only. Similarly, prolonged COVID-19 lockdowns and social distancing restrictions in Victoria impeded the completion of paper surveys and face-to-face recruitment activities in that state. Other variations in the recruitment strategies reflected the preferences, priorities, and capacities of the local partner organizations. 

### 2.1. Recruitment to the Online Survey 

**Figure 1:**
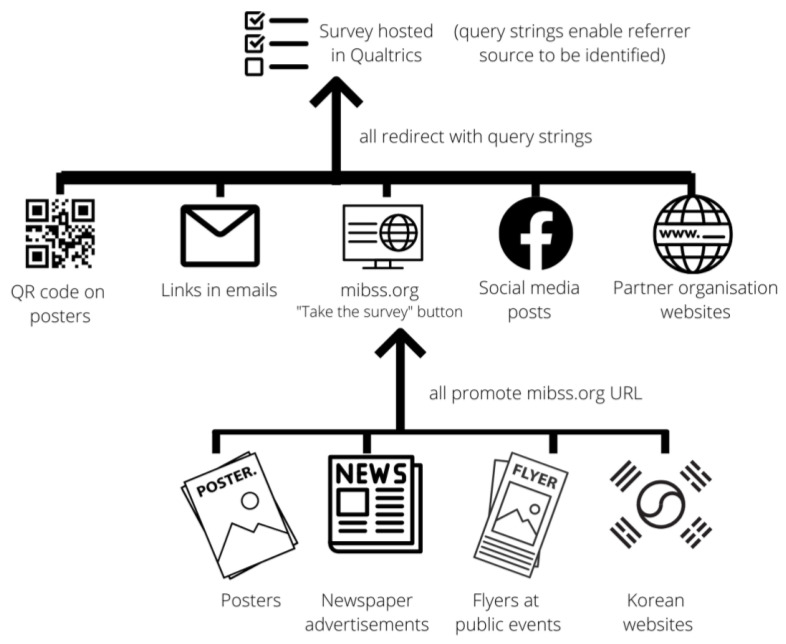
Pathways for online recruitment.

Figure 1 summarizes the architecture for the various streams for online recruitment. The online survey was built and hosted on the Qualtrics platform [17]. A link to the Qualtrics survey was embedded in a prominent ‘Take the Survey’ button on the home page of the branded project website. The branded website uniform resource locator (URL)—www.mibss.org (accessed on 26 May 2021)—was advertised in four ways:

1. Through print and online media advertisements published in (a) Korean-language newspapers (*Vision Weekly* and *The Sunday Weekly*; collective readership, 11,500) on 2 October 2020, (b) *SS Newspaper* (readership, 5000) on 6 October 2020, (c) *AVN Newspaper* (readership, 5000) on 30 October 2020, (d) *Viet Queensland News Online* on 7 October 2020, (e) *Asian Community Newspaper* (*ACNews*), and *Queensland Asian Business Weekly* on 24 September 2020 (collective readership, 35,000);
2. On a flyer handed out by project officers at public events/locations (namely, to individuals who expressed interest in the survey but were not able or willing to complete the paper version at that time);
3. On a poster that was displayed in public places frequented by the target population. The poster also featured a quick-response (QR) code link; and
4. By project officers attempting to recruit individuals by phone call or text message.

Additionally, a project page was created on both Facebook and Twitter. Between 7 September 2020 and 4 May 2021, 31 Facebook posts containing links to both the Qualtrics survey and the project website were published, including four paid advertising boosts (each four to five days in duration) targeting users attending Australian urban tertiary educational institutions in an effort to reach international students. Key partners were ‘tagged’ in posts and were encouraged to share/repost content. The number and demographics of the people who were reached by and engaged with each post were determined using the ‘Page Insights’ function in Facebook. A Korean project officer in South Australia also took the initiative to post survey details in private Facebook groups, namely International Student Support (the University of Adelaide); Koreans in Sydney; Koreans in Melbourne; Ethnic Communities Council of Queensland BBV-Korean; Hojutimes; Korean Community in Australia; Asians in Sydney, Melbourne, and Brisbane; and Asian Around Sydney. Data on reach and demography are not available for posts in these external group pages. 

Fifteen posts were published from the project Twitter account from September 2020 until January 2021; however, a review of the social marketing strategy revealed that the Twitter posts had attracted only one survey respondent in that time, and a decision was therefore made to discontinue the use of Twitter for project recruitment. 

Finally, partner organizations, funders, and other collaborating organizations, including research institutes and service providers, were encouraged to email the survey link to members of their networks. Three organizations also hosted links to the survey on their own websites (Western Australian AIDS Council (WAAC), Western Australian Office of Multicultural Interests, and Multicultural HIV and Hepatitis Service New South Wales), and a Korean project officer in South Australia posted information about the survey (with links to the project website) on five Korean-Australian websites, namely Adelaide Focus (www.adelaidefocus.com, accessed on 21 December 2020), Adelaide.co (www.adelaide.co.kr, accessed on 28 November 2020), Adelaide4989.com, accessed on 28 November 2020, Hojubada.com, accessed on 18 December 2020, MissyAUS (https://cafe.naver.com/missyaus, accessed on 17 February 2021), and Kangaroo (https://cafe.naver.com/kangaroo, accessed on 16 December 2020). 

Qualtrics software enables data to be passed into a survey using a query string. Specific strings were created for each referring source (e.g., project website, Facebook, Twitter, QR code, partner email, and partner website), and these were appended to the relevant Qualtrics survey URL to enable the software to capture referral sources for each survey participant. For example, survey links sent to the target populations in emails from the Ethnic Communities Council of Queensland all ended with the string ‘Source=ECCQemail’, and surveys accessed from the www.mibss.org website ended with ‘Source=mibss’. 

It was not possible to ascertain whether a respondent visited www.mibss.org and completed the survey as a result of seeing the URL in a newspaper advertisement, flyer, poster, Facebook post, or Korean website (Figure 1). However, some inferences about sources of referral were made by graphically mapping (1) the dates on which www.mibss.org was promoted through various media against (2) the dates on which participants accessed the survey via www.mibss.org, as well as (3) the demographic characteristics of those participants. For instance, if a large number of South Korean participants accessed the survey via www.mibss.org on the day that the URL was posted on a South Korean website (and if there were no other promotions on that day), it is open to infer that those participants were referred from the South Korean website post.

All online survey participants were offered the opportunity to enter a draw to win a AUD 200 voucher prize by clicking on a link that redirected them to a separate form; this ensured that no identifying personal information could be linked to individual survey responses. 

### 2.2. Recruitment to the Paper Survey 

Due to separate funding and administrative arrangements, respondents in Queensland who commenced the paper version of the survey were provided a gratuity (AUD 30 value). In February 2021, ethics approval was granted to enable respondents to the paper survey in all other states to receive a gratuity (AUD 15 value) in response to feedback that respondents would value some recognition of the time taken to complete the survey; prior to this, respondents in those states were only provided with an opportunity to enter a prize draw. The amount reflected both the available budget and concerns about whether the practice of offering $30 per participant would be financially sustainable if the survey were repeated periodically on larger samples. 

With respect to the provision of gratuities, the Curtin University Human Research Ethics Office guidelines for payments to research participants were followed; these guidelines are consistent with the National Statement on Ethical Conduct in Human Research, which notes that “the customs and practices of the community in which the research is to be conducted” are relevant considerations in decision making regarding payments [18,19]. The decision to provide gratuities in this study was made in consultation with partner organizations with experience working with migrant communities in Australia. 

Where practical in the context of COVID-19, project officers attended public events and venues in South Australia, Western Australia, and Queensland to invite people to complete the paper version of the survey. A full list of the events/venues attended is presented in the Results section. Project officers were also encouraged to approach eligible individuals in their own personal or organizational networks to complete the paper survey. 

Respondents were instructed to seal their completed paper surveys in an unmarked envelope to preserve confidentiality, and these sealed envelopes were then sent to the project team for manual data entry. 

### 2.3. Data, Variables, and Analysis

Survey data collected in Qualtrics were analyzed with Stata Statistical Software Release 16 [20]. Respondents were excluded from the dataset if they did not meet eligibility criteria. Respondents whom Qualtrics indicated had made less than 25% progress were also excluded from analysis on the assumption that these respondents were exploring the survey without intending to formally participate (for instance, some stakeholders browsed the survey contents before forwarding to potential respondents). 

The categorical variables set out in Table 2 were created. Descriptive statistical analyses (frequency and percentage) were used to determine the demographic characteristics of the sample dataset, in addition to the numbers and proportions of respondents recruited to complete the paper and online surveys and sources of referral for online respondents. Logistic regression analyses were run to predict online participation generally according to region of birth, state of residence, age, gender, years in Australia, and sexual identity, using dummy coding for multilevel categories. The significance threshold was set at 0.05. 

Data on paper survey recruitment at public events were collected from records provided by project officers based on a template provided by the researchers (Appendix A). These records detailed the date and nature of the event, the number of recruitment attempts, the number of ineligible persons identified, the number of refusals (with reasons for refusal and any observations on survey refusal trends), and the number of respondents recruited. Project officers were also asked to record in-person invitations to participate in their personal or organizational networks (Appendix B). Frequencies were calculated, along with participation rates, using the number of respondents as the numerator and the number of people approached (excluding those known to be ineligible) as the denominator. 

Differences between groups (e.g., gender, region of birth, and age) were tested for statistical significance (at a significance threshold 0.05) using the chi-squared test or Fisher’s exact test if more than 20% of cells had expected values of less than five.

A binary variable (pre-March 2021/post March 2021) was created to indicate whether a paper survey was completed before gratuities for paper surveys were introduced in all states, and descriptive statistics were used to show pre/post comparisons for each state. 

Finally, contemporaneous records from partner organizations regarding recruitment (e.g., emails sent to the Project Coordinator) and open text responses to the ‘feedback’ item in the survey were also reviewed for evidence of other barriers to recruitment. Two themes emerged from thematic analysis, namely concerns about stigma and confidentiality. Illustrative quotations are presented. 

## 3. Results

### 3.1. Sample Characteristics 

The target sample goal was achieved, and 1454 respondents were recruited to the study over approximately nine months (September 2020–May 2021) during the COVID-19 pandemic. Due to the unique experience of the COVID-19 pandemic, in Victoria, an additional 35 respondents were recruited for the paper survey after recruitment was concluded in other states and the online survey was deactivated; however, these respondents are not included in this analysis and are instead treated as a distinct recruitment period. 

The characteristics of the national sample are summarized in Table 3. With respect to self-reported region of birth, 36.66% (n = 533) of the sample was born in a South-East Asian country, 29.02% (n = 422) was born in a North-East Asian country, and 24.62% (n = 358) was born in Sub-Saharan Africa; the remaining 9.70% (n = 141) of the sample did not report a country of birth or provided an illegible response. Just over half (54.54%; n = 793) of the sample were aged 18–39 years of age, and the majority (59.42%; n = 864) identified as women. More than three-quarters of the sample had lived in Australia for fewer than 20 years (75.72%; n = 1101). Same-sex attraction (either exclusively or in addition to attraction to other sexes) was reported by 7.36% (n = 107) of the sample. 

### 3.2. Online Survey Recruitment

The online version of the survey was completed by 28.27% (n = 411) of the sample, and of these, the majority (65.21%; n = 268) elected to enter the draw to win the AUD 200 voucher. The data in Table 4 identify the characteristics of online survey participants. Statistically significant predictors of online completion included identifying as a female, being born in North-East Asia, being aged 40–49 years, reporting same-sex attraction, and residing in Victoria or South Australia.

Of the online completions, 105 (25.55%) were referred from emails sent by partner organizations promoting/inviting people to participate in the study, 21 (5.11%) were referred from the poster QR codes, 8 (1.95%) were referred from the websites of research partner organizations, and 1 (0.24%) was referred from Twitter. 

**Figure 2:**
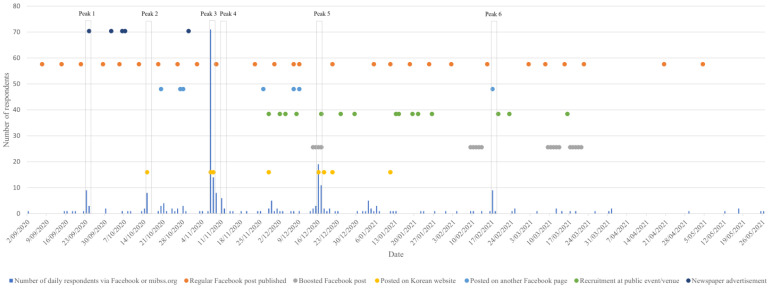
Number of daily respondents to the online survey (referred via Facebook or mibss.org) mapped against date and method of promotion.

The remainder of online completions (67.15%; n = 276) were from either Facebook or from the project branded website (we do not distinguish between the two, as Facebook posts included both a unique link to the survey and a link to the branded website to enable people to find more information before participating). Figure 2 shows peaks in daily survey completions relative to key dates on which the online survey was promoted and the methods of promotion. An analysis of the demographics of respondents during the six peaks provides some insights into the relative effectiveness of the different methods of promotion (Table 5). Peaks are defined here as a period of two consecutive days, commencing on a day when the number of respondents exceeds the number of respondents on the previous day by at least five. In peak 3, which coincided with posts on Korean-Australian websites, respondents were statistically significantly more likely to be Korean compared to respondents referred from Facebook and mibss.org during other time periods. Only one peak (peak 5) coincided with a paid Facebook advertising boost; the boost targeted Western Australian audiences, and five Western Australian respondents were recruited during this peak. 

### 3.3. Paper Survey Recruitment 

Project officers in Queensland and Western Australia provided data on attempts to recruit participants to complete paper surveys at public events/locations (no public event/location recruitment occurred in New South Wales due to funding or Victoria due to COVID-19) (Table 6 below). In total, 155 respondents were recruited through this method, with the largest proportion being recruited from a World AIDS Day event in Queensland (31.61%; n = 49) and a series of religious fellowship group meetings in Western Australia (29.03%; n = 45).

Aside from recruitment at public events/venues, the remainder of the paper survey respondents were recruited through direct approaches (face-to-face, email, telephone, and text message) to project officers’ individual and organizational networks. 

**Figure 3:**
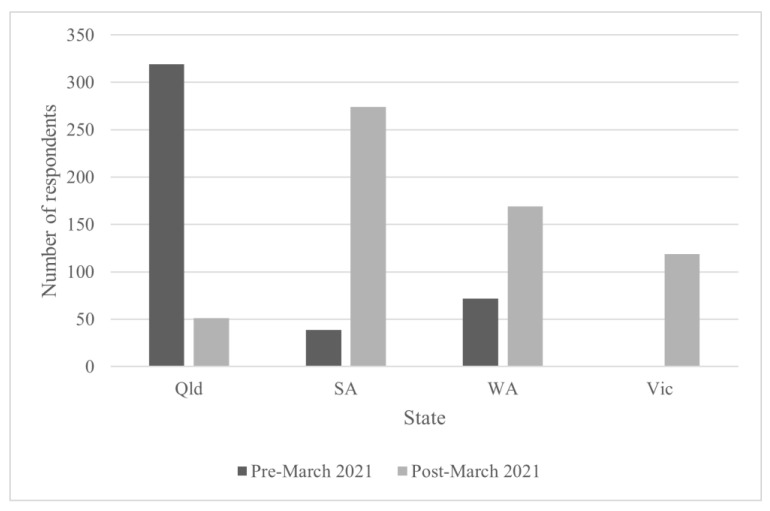
Number of paper survey respondents by state pre- and post March 2021.

Figure 3 shows that before March 2021 (when AUD 15 gratuities were introduced for paper survey respondents in Victoria, South Australia, and Western Australia), a total of 111 respondents had completed the paper survey in those states, compared to 319 respondents who had completed a paper survey in Queensland (which offered AUD 30 gratuities from the commencement of the study). After March 2021, 562 paper survey respondents were recruited in Victoria, South Australia, and Western Australia (in Queensland, there were fewer respondents after March compared to before March, as the Queensland sampling quota was reached earlier than in other states). 

### 3.4. Participation Rates 

Although the overall participation rate for public event/location recruitment was low (36.99%; n = 155), some venues/events resulted in higher rates of participation; these included a World AIDS Day Event in Queensland (94.23% participation rate; n = 49), a sexually transmissible infection (STI) and human papillomavirus (HPV) vaccination workshop (100% participation rate; n = 13), and a religious fellowship meeting (54.05%; n = 20, although other fellowship meetings resulted in lower participation rates). The participation rates do not include individuals who took a flyer containing a link to the branded project website and may have completed the survey online at a later time (see Section 3.2 above). The most common reasons for refusal to participate at public events/locations were “too busy” (n = 95; 37.84%) and “took link for completion at later date” (n = 93; 37.05%). 

Not all efforts to recruit individuals from individual/organizational networks were documented by project officers; the implications of this will be set out in the Discussion. Of the available records that documented numbers and types of recruitment attempts (other than those at public events/venues), the majority recorded one contact attempt (69.14%; n = 242), 22.57% (n = 79) recorded two contact attempts, and 8.29% (n = 29) recorded three contact attempts. The overall participation rate was 82.75%, as shown in Table 7, and attempts that involved at least one face-to-face contact had statistically significantly higher rates of participation than those that involved only contact by email, telephone, or text message. With respect to recruitment outcomes (survey completed versus refused/no response), no statistically significant differences were observed with respect to gender, age, or region of birth (survey completed by: 83.95% of males, 81.56% females, *p* = 0.561; 79.45% 18–29 years, 82.80% 30–39 years, 83.67% 40–49 years, 86.67% 50–59 years, 79.17% over 65 years, *p* = 0.862; 83.33% NEA, 77.78% SEA; 83.12% SSA, *p* = 0.723)). The most common reasons for refusal to participate in response to personal/organizational network recruitment attempts were “too busy” (n = 14; 29.17%) and “uncomfortable” (n = 16; 33.33%).

It is not possible to discern the number of eligible individuals who were exposed to invitations to participate contained on posters, in Facebook posts, in newspaper advertisements, and on other websites.

### 3.5. Documented Barriers to Participation 

Some data suggest that the name of the project—the Migrant Blood-borne Virus and Sexual Health Survey—may have been a barrier to recruitment for some participants, notwithstanding that no issue was raised during the survey pretesting phase [15]. Two emails received during data collection suggested that the name could be interpreted as implying that migrants were ‘carriers’ of or uniquely affected by STIs and BBVs. The link between ‘migrants’ and ‘viruses’ was considered particularly problematic during the COVID-19 pandemic:

*A person from the Philippines] said we should not use “Migrant Blood-borne Virus and Sexual Health Survey” as she thinks it meant migrants brought these diseases here. It would cause more stigma due to the current COVID-19 situation. She said we should use “Blood-borne Virus and Sexual Health Survey for Migrants”. She said she could not pass the survey to others because of this.*

(personal communication, email received from Queensland partner organization, 15 September 2020).

*I am concerned about this research title Migrant Blood-borne Virus and Sexual Health Survey, it’s particularly sensitive under the current context that Asian communities are stigmatized and there is “I am not Virus Campaign”*

(personal communication, email received from New South Wales stakeholder, 21 September 2020).

Additionally, responses to an open text question inviting survey participants to provide feedback/comments on the survey indicated some uncertainty with respect to whether project officers who administered and collected the paper surveys would be able to view their responses:

*Is this survey confidential for people who are collecting it?*

(Sub-Saharan African-born respondent, aged 40–29).

*We don’t know how you keep the confidentially [sic] of this survey, since I got this survey from a girl I am not close with.*

(South-East Asian-born respondent, aged 18–29 years).

While the feedback came from participants who had completed the survey, it is plausible that similar concerns were held by those who refused to participate but did not articulate a reason.

## 4. Discussion

Our experiences demonstrate that, even in the context of challenges associated with the COVID-19 pandemic, it is possible to recruit a large sample of African and Asian-born migrants to participate in a sensitive (SHBBV) survey through a community-partnership approach involving a suite of engagement strategies. Among the range of recruitment strategies adopted, the largest number of participants were recruited through direct invitations from project officers with migrant backgrounds working within multicultural partner organizations. This is consistent with Bonevski and colleagues’ systematic review, which found evidence that recruitment within socioeconomically disadvantaged groups is improved through the involvement of community groups and culturally trained fieldworkers, peers, or local researchers [4]. Similarly, in the context of a clinical trial in a multicultural population, MacEntee and colleagues found that “direct recruitment was much more effective than an indirect approach for recruiting subjects from ethnic minorities”, with contact persons from local community centers assisting to cultivate trust and understanding of the project [21] (p. 379). 

We encourage researchers to consider our model of funding local organizations with strong migrant community networks (especially those with experience working on the health issues being researched) to employ project officers to drive recruitment. The model aligns with best-practice ethical recommendations around the need to develop meaningful partnerships and engage stakeholders, community leaders, and community members in the design and conduct of migrant health research [22,23]. Similar community partnership approaches to recruitment have been applied successfully in the context of other SHBBV surveys in migrant contexts [24].

Not all forms of direct recruitment by project officers with migrant backgrounds were equally successful. Face-to-face recruitment through organizational/personal networks was more successful than in-person recruitment at public venues and events. As was demonstrated in Bonevski and colleagues’ review, trust plays a key role in decision making around research participation [4]. Being approached in a public place by an unknown person (especially in the context of a pandemic) and invited to participate in a survey on a sensitive subject is likely to evoke more suspicion than being privately approached by a familiar individual, or an individual with links to a familiar organization. There were some notable exceptions; for instance, recruitment at a Worlds AIDS Day event hosted by the Queensland partner organization resulted in a 94.24% participation rate. However, given the heterogeneity within migrant communities, care must be taken in selecting an appropriate range of public events/venues in which to undertake recruitment. SHBBV survey research conducted in migrant communities before the COVID-19 pandemic suggests that other options for public recruitment sites/events include multicultural festivals and celebrations, as well as soccer games involving culturally and linguistically diverse teams [25]. 

In addition to drawing upon their networks to recruit participants for the paper survey, project officers also used their knowledge of the source population to pursue innovative methods of online recruitment. The most obvious example of this was the decision to post survey links on websites specifically developed for migrant communities, such as the Korean-Australian websites Adelaide Focus and MissyAUS. Whereas the literature on the use of this recruitment strategy for migrants is limited, one Australian study noted similar successes using Chinese social network sites such as Oursteps, Ozyoyo, and Freeoz [26]. The fact that this recruitment method was driven by an individual local project officer in our study meant that a unique string query was not created to enable participants referred from these sites to be precisely quantified. Future researchers should consider including this method of recruitment in their study protocols and create unique string queries for survey URLs posted on these sites; this will enable researchers to collect empirical evidence on the effectiveness of recruitment via these websites and online communities in a more systematic way. 

In comparison, the use of mainstream social media was less effective for recruitment; notably, only one modest peak in online survey recruitment coincided with a paid Facebook advertising boost (Figure 2). This is consistent with a Dutch study of Turkish migrants, which failed to recruit any study participants through a Facebook advertisement [27]. Since 2020, it has not been possible to use “multicultural affinity segments” (e.g., Hispanic) to target advertising content to specific ethnic groups (the ability to exclude ethnic groups from advertising strategies has been prohibited since 2017) [28,29]. Whereas these changes to Facebook’s advertising policies are a justified response to discriminatory commercial advertising practices, they also affect the ability of researchers to use Facebook to recruit from historically underrepresented populations. Consequently, more research is needed about effective ways of engaging with culturally and linguistically diverse populations via Facebook and other social media platforms. Although tagging partner organizations (e.g., multicultural organizations and service providers) and encouraging them to share posts with their own networks helped to bring the content to the attention of eligible participants, promoting content “in this manner can quickly result in saturation” [30] (p. 7). Consideration must also be given to the risk of stigmatizing population groups by tagging them in posts relating to communicable diseases.

Fewer people were recruited to the online survey compared to the paper survey; nevertheless, the data we have presented on the results of our online survey recruitment methods represent an important contribution to the literature, given that a recent scoping review on modes of administering SHBBV surveys in migrant populations found only four studies in which online participation was discussed [31]. Online recruitment methods played an important complementary role in our sampling strategy. For instance, same-sex attraction was a statistically significant predictor of online completion in this study; this suggests that online recruitment may increase sample diversity and/or increase the willingness of respondents to provide truthful responses to culturally/socially sensitive questions about sexual identity due to the perception that online completion is more private. Other studies have revealed that “greater anonymity and ease of participation with online surveys compared to face-to-face data collection may increase the probability that individuals in same sex unions and same sex couples will participate in studies” [32] (pp. 100–101). However, there are examples of same-sex-attracted migrants participating in print SHBBV surveys in Australia through recruitment at gay community events, sex-on-premises venues, and testing clinics, as well as through the involvement of same-sex-attracted peer researchers [33]. 

Feedback from some survey respondents in our study suggests that concerns about anonymity associated with in-person recruitment may not be limited to same-sex-attracted people. In the context of a survey on a sensitive subject (in this case, sexual health knowledge and behaviors), a small number of individuals from migrant backgrounds reported being wary of completing the survey and handing it back to project officers from local multicultural organizations. Although using peers or local researchers can aid recruitment in the ways outlined above, researchers also need to be mindful that ‘insiders’ can be regarded with suspicion due to concerns about confidentiality and other intra-group dynamics [34,35]. Paper survey respondents were encouraged to seal their completed surveys in blank envelopes before handing them back to project officers, but other strategies should also be considered to overcome concerns about privacy. Strategies that would benefit from further research in migrant contexts include (1) providing prepaid, addressed envelopes to enable respondents to mail completed surveys directly to the lead researchers, (2) the use of tamperproof/self-adhesive seals or envelopes, and (3) clearer communication around who will be permitted to view the data, as well as ethical obligations around confidentiality. 

Feedback also revealed that the survey name may have impacted recruitment efforts in so far as it implied, to some people, that sexually transmissible infections and blood-borne viruses only affect (or are transmitted by) migrants. This feedback is consistent with a study on the feasibility of engaging migrants living in Germany in health interview surveys [36]. Zeisler and colleagues found that some migrants refused to participate in their study because they did not understand the study objectives or were critical of the focus on migrants (“Are there diseases that only affect special population groups?”) [36]. While our messaging through social media and promotional videos emphasized that sexually transmissible infections and blood-borne viruses affect all groups, the message may have been undermined by incorporating the term “migrant” in the project name. As suggested in the feedback quoted in Section 3.5, the way the project name was interpreted by some individuals may have been influenced by the fact that recruitment coincided with an increase in stigmatization of migrant communities in relation to the COVID-19 pandemic [37]. Just as the World Health Organization has recommended that disease names should avoid references to specific geographic locations, cultures, and populations, our experience suggests that it would be advisable for researchers to avoid adopting project names that could be used to create a negative association between migrants and communicable diseases [38]. Future iterations of this survey will be rebranded accordingly, with community consultation. 

We found high acceptance of gratuities in our sample of South-East Asian, North-East Asian, and Sub-Saharan African migrants living in Australia. Almost two-thirds of online respondents elected to enter a draw to win a voucher valued at AUD 200. Moreover, as indicated in the Materials and Methods section, due to funding arrangements, originally, only Queensland participants were offered a gratuity for completing the paper version of the survey; however, gratuities for paper-survey participants were introduced in Victoria, South Australia, and Western Australia in response to feedback from partner organizations and early survey participants. 

Although the number of participants increased following the introduction of gratuities in South Australia, Western Australia, and Victoria, it is not possible to discount the role of other factors, including (a) the impact of COVID-19-related public health orders pre-March 2021, which prohibited large public gatherings in some areas in response to localized outbreaks; (b) specific organizational issues affecting partner organizations responsible for recruitment in each state, including the presence of competing work priorities and issues associated with employing and retaining appropriate staff to facilitate survey recruitment; (c) public willingness to engage in research in earlier stages of the pandemic; and (d) the effects of stigmatizing language and practices toward migrant communities in relation to COVID-19, particularly at the commencement of the pandemic. Nevertheless, the findings are consistent with those of a recent meta-analysis of randomized controlled trials investigating the effect of monetary incentives on survey response rates. Jia and colleagues found that final relative response rates increased by approximately 20% when monetary incentives were offered compared to no incentive [39]. Moreover, the meta-analysis of results from trials in which a dose–response relationship was tested revealed that “monetary incentives between US$10 and US$15 have the maximum effect on the final response rate” [39]. The fact that South Australia (where gratuities provided were worth AUD 15) managed to recruit a similar number of participants as Queensland (which provided gratuities valued at AUD 30) suggests that recruitment targets can be met with more modest gratuities; this finding is relevant for researchers seeking to administer large but modestly funded population surveys. 

There remains a debate about the appropriateness of providing gratuities to survey participants. Whereas systematic reviews have identified little evidence that gratuities have any significant effect on the quality of survey responses, sample composition, and response distributions, it is acknowledged that more research is needed [40]. Moreover, ethical questions remain as to whether the provision of gratuities to participants amounts to coercion or undue influence [41]. Our decision to provide gratuities in this instance was informed by community consultation and “arguments that non-payment, or insufficient payment, may also be unethical” [42]. The existence of “research fatigue” in some migrant communities has been documented and is associated with calls for more tangible benefits to research participants based on the principle of reciprocity [43].

The most obvious shortcoming in terms of recruitment related to the fact that only 34.49% of total respondents identified as men. Although the underrepresentation of men in survey research on sexual health/behaviors is not uncommon [44,45,46,47,48], there are some examples from migrant communities in which samples were more evenly distributed by gender [25,49]. It is not possible to empirically ascertain the reason for the low proportion of men in our study. However, the literature contains a number of examples of how representation of men can be increased; these include encouraging women to invite men from their own networks to participate and tailoring social media content and recruitment materials to men (e.g., by using images of men) [50,51]. Additionally, it is possible that recruiting more men as project officers/peer researchers to facilitate data collection may assist in overcoming cultural sensitivities around intergender interactions, particularly on the subject of sexual health knowledge/behaviors [34]. There is also evidence to suggest that peer researchers tend to recruit respondents whose gender, age, and region of birth are similar to their own [51]. 

Another major limitation relates to the fact that protocols around keeping records of recruitment attempts were not maintained by all project officers employed by local partner agencies (Appendix A and Appendix B). Consequently, our observations are necessarily limited to the available data, and the possibility that the missing data may be materially different to the available data cannot be discounted. Although this is a major shortcoming, it also provides an accurate insight into the challenges associated with devolving data collection responsibilities to lay researchers who, despite training, may not be as familiar with the importance of scientific rigor. Other studies have documented instances in which the advantages associated with involving peer/lay researchers in studies have been accompanied by challenges associated with adherence to research best practice [52,53]. Our findings on the importance of involving local lay researchers and community organizations and being accommodating of their suggestions demonstrate that the solution is not for professional researchers to either retake or tighten their control of data collection processes. Rather, more work needs to be done to build capacity outside of academia so that organizations and individuals closest to the populations being studied are better equipped to collect data in a way that is scientifically rigorous. Solutions include investigating ways of improving training in research methods for non-academic audiences, exploring innovative ways of simplifying data collection tools and processes, and ensuring that community organizations are appropriately funded to embed research engagement staff into their workforce. 

## 5. Conclusions

Far from being ‘hard to reach’, our research shows that migrant populations can be engaged in survey research on a sensitive topic, even in the face of recruitment challenges associated with the COVID-19 pandemic. Although our study of recruitment strategies was not designed to be experimental, we demonstrated how efforts to document recruitment strategies and outcomes can assist future researchers to engage with migrant populations in a manner that is both more efficient and culturally sensitive. In addition to confirming existing research on the importance of strong partnerships with community members and organizations to build trust and legitimacy, our research has also revealed novel avenues for recruitment that warrant further attention, such as promotion through websites that specifically serve migrant communities. Our findings also demonstrate the challenges associated with Facebook advertising as a recruitment strategy for migrants, and we caution researchers against investing scarce project resources into this method in the absence of new evidence of effectiveness. We encourage more researchers to document and publish their experiences of recruiting members of migrant populations in an effort to build the evidence base and strengthen investment to redress the underrepresentation of this priority group in health and medical research. 

## Figures and Tables

**Table 1:** Summary of recruitment methods in each participating Australian state.

Method	Western Australia	South Australia	Victoria	New South Wales	Queensland
Poster QR codes	Yes	Yes	Yes	Yes	Yes
Email invitations	Yes	Yes	Yes	Yes	Yes
Social media posts	Yes	Yes	Yes	Yes	Yes
Facebook advertisements	Yes	Yes	Yes	Yes	Yes
Newspaper advertisements	No	No	No	No	Yes
Posts on local websites	Yes	Yes	No	Yes	Yes
Attending public venues	Yes	Yes	No	No	Yes

**Table 2:** Summary of variables used in statistical analysis.

Variables	Categories	Source Data
Region of birth	NEA; SEA; SSA; not reported ^1^	Based on responses to survey item ‘country of birth’
State of residence	WA; Qld; SA; Vic; NSW; not reported/other ^2^	Based on reported postcode, information in source query string, or state paper surveys were collected from
Age in years	18–29; 30–39; 40–49; 50–59; 60+; not reported	Based on responses to survey item ‘age’
Gender	Male; female; not reported or other	Based on responses to survey item ‘own gender’
Years in Australia	≤9; 10–19; 20–29; 30+; not reported	Based on responses to survey item ‘time in Australia’
Sexuality	Heterosexually attracted only; same-sex attraction; not reported/other	Based on matching responses to survey item ‘own gender’ and ‘gender of those to whom sexually attracted’
Survey type	Online; paper	Based on information in source query string
Online referral source	WA sexual health organization website; WA multicultural organization website; Korean-Australian website; NSW Government website; mibss.org; email from WA partner; email from Qld partner; email from Victorian partner; email from NSW partner; email from SA partner; QR code; Twitter; Facebook	Based on information in source query string

^1^ NEA = North-East Asia; SEA = South-East Asia; SSA = Sub-Saharan Africa. ^2^ WA = Western Australia; Qld = Queensland; SA = South Australia; Vic = Victoria; NSW = New South Wales.

**Table 3:** Number of respondents according to region of birth, state of residence, age, gender, time in Australia, and sexuality.

Place of Birth	State of Residence						Age (Years)						Gender			Length of Time in Australia (Years)					Sexuality		
	WA	QLD	SA	VIC	NSW	N/A	18–29	30–39	40–49	50–59	60+	N/A	Male	Female	N/A & Other	≤9	10–19	20–29	30+	N/A	Hetero-Sexual Only	Same-Sex Attraction	N/A & Un-defined
**SSA (n = 358)**	**42**	**159**	**132**	**20**	**3**	**2**	**110**	**116**	**81**	**37**	**11**	**3**	**163**	**184**	**11**	**121**	**198**	**14**	**6**	**19**	**306**	**28**	**24**
Eastern ^1^ (n = 267)	37	124	88	18	0	0	83	91	60	25	8	0	122	136	9	84	159	9	3	12	232	17	18
Western ^2^ (n = 53)	1	17	30	1	2	2	18	11	9	10	3	2	24	28	1	21	21	4	1	6	43	5	5
Central ^3^ (n = 22)	0	12	10	0	0	0	8	7	5	1	0	1	13	9	0	10	12	0	0	0	19	3	0
Southern ^4^ (n = 16)	4	6	4	1	1	0	1	7	7	1	0	0	4	11	1	6	6	1	2	1	12	3	1
**SEA (n = 533)**	**213**	**134**	**95**	**83**	**6**	**2**	**127**	**117**	**115**	**83**	**82**	**9**	**193**	**329**	**11**	**220**	**136**	**54**	**105**	**18**	**445**	**43**	**45**
Cambodia (n = 27)	0	4	23	0	0	0	12	6	7	1	1	0	16	10	1	18	6	0	3	0	17	6	4
Indonesia (n = 165)	141	12	3	7	1	1	31	37	46	34	15	2	50	115	0	70	50	21	18	6	154	8	3
Malaysia (n = 64)	25	20	7	11	1	0	19	14	9	7	15	0	24	38	2	24	19	4	14	3	47	7	8
Myanmar (n = 27)	2	22	3	0	0	0	8	5	4	4	6	0	11	15	1	14	4	3	5	1	21	2	4
Philippines (n = 104)	12	10	40	42	0	0	22	25	17	18	20	2	30	71	3	46	18	7	28	5	83	8	13
Singapore (n = 33)	16	8	2	7	0	0	7	3	5	7	10	1	8	25	0	9	9	6	8	1	29	4	0
Thailand (n = 22)	2	5	2	13	0	0	4	10	8	0	0	0	9	12	1	8	11	1	2	0	14	3	5
Vietnam (n = 88)	14	53	15	2	3	1	23	15	19	12	15	4	43	42	3	31	18	11	26	2	76	5	7
Other (small cells combined) (n = 3)	1	0	0	1	1	0	1	2	0	0	0	0	2	1	0	0	1	1	1	0	2	0	1
**NEA (n = 422)**	**37**	**125**	**144**	**70**	**37**	**9**	**118**	**152**	**104**	**28**	**18**	**2**	**113**	**307**	**2**	**224**	**140**	**34**	**14**	**10**	**366**	**31**	**25**
Mainland China (n = 164)	13	49	75	25	0	2	70	41	27	16	8	2	63	100	1	108	33	11	6	6	139	11	14
Hong Kong/Macau (n = 14)	5	2	5	2	0	0	4	6	1	2	1	0	3	10	1	9	5	0	0	0	11	2	1
Taiwan (n = 41)	6	20	11	3	0	1	5	20	8	2	6	0	11	30	0	21	9	3	6	2	33	3	5
Japan (n = 23)	1	3	16	1	0	2	4	5	13	1	0	0	3	20	0	8	13	1	0	1	20	3	0
Korean peninsula (n = 180)	12	51	37	39	37	4	35	80	55	7	3	0	33	147	0	78	80	19	2	1	163	12	5
**Unknown (n = 141)**	**16**	**34**	**46**	**5**	**4**	**36**	**21**	**32**	**11**	**18**	**5**	**54**	**36**	**44**	**61**	**23**	**39**	**2**	**9**	**68**	**65**	**5**	**71**
**TOTAL (n = 1454)** **(%)**	**308** **(21.18)**	**452** **(31.09)**	**417** **(28.68)**	**178** **(12.24)**	**50** **(3.44)**	**49** **(3.37)**	**376** **(25.86)**	**417** **(28.68)**	**311** **(21.39)**	**166** **(11.42)**	**116** **(7.98)**	**68** **(4.68)**	**505** **(34.73)**	**864** **(59.42)**	**85** **(5.85)**	**588** **(40.44)**	**513** **(35.28)**	**104** **(7.15)**	**134** **(9.22)**	**115** **(7.91)**	**1182** **(81.29)**	**107** **(7.36)**	**165** **(11.35)**

^1^ Eastern Sub-Saharan Africa is defined here as Tanzania, Kenya, Uganda, Rwanda, Burundi, Sudan, South Sudan, Eritrea, Ethiopia, Somalia, Comoros, Mauritius, Seychelles, Mozambique, Madagascar, Malawi, Zambia, and Zimbabwe. ^2^ Western Sub-Saharan Africa is defined here as Benin, Burkina Faso, Cape Verde, The Gambia, Ghana, Guinea, Guinea-Bissau, Ivory Coast, Liberia, Mali, Mauritania, Niger, Nigeria, Senegal, Sierra Leone, and Togo. ^3^ Central Sub-Saharan Africa is defined here as Angola, Cameroon, Central African Republic, Chad, Democratic Republic of Congo, Republic of Congo, Equatorial Guinea, Gabon, Sao Tome, and Principe. ^4^ Southern Sub-Saharan Africa is defined here as Botswana, Eswatini, Lesotho, Namibia, and South Africa.

**Table 4:** Characteristics of participants completing the online survey.

Correlate	Proportion (%) of Total Sample (n = 1454)	Proportion of Online Sample (n = 411)	Odds Ratio	95% Confidence Interval	*p*-Value
**Total online completion (n = 411)**	28.27	-	-	-	-
**Gender**					
Male	5.98	21.17	-	-	
Female	18.71	66.18	2.21	1.68–2.90	<0.001
Invalid/missing response	3.58	12.65	7.57	4.62–12.40	<0.001
**Region of birth**					
North-East Asia	14.37	50.85	-	-	
South-East Asia	6.46	22.87	0.22	0.16–0.29	<0.001
Sub-Saharan Africa	3.78	13.38	0.18	0.13–0.26	<0.001
Invalid/missing response	3.65	12.90	0.61	0.42–0.91	0.014
**Age (years)**					
18–29	6.46	22.87	-	-	
30–39	8.60	30.41	1.28	0.94–1.76	0.118
40–49	7.02	24.82	1.46	1.05–2.04	0.025
50–59	1.58	5.60	0.48	0.29–0.79	0.004
60 and older	1.31	4.62	0.59	0.34–1.01	0.056
Invalid/missing response	3.30	11.68	7.20	4.07–12.75	<0.001
**Sexual attraction**					
Heterosexual only	20.84	73.72	-	-	
Same sex	2.75	9.73	1.73	1.15–2.62	0.009
Invalid/missing response	4.68	16.55	2.03	1.45–2.85	<0.001
State *					
Queensland	5.84	19.95	-	-	
South Australia	7.40	25.30	1.50	1.08–2.08	0.015
Western Australia	4.77	16.30	1.25	0.87–1.80	0.219
Victoria	4.20	14.36	2.23	1.51–3.31	<0.001
**Length of time in Australia (years)**					
Fewer than 10	11.69	41.36	-	-	
10–19	8.73	30.90	0.81	0.62–1.06	0.122
20–29	2.20	7.79	1.09	0.69–1.72	0.701
30 or more	1.72	6.08	0.56	0.35–0.90	0.017
Invalid/missing response	3.92	13.87	2.42	1.61–3.63	<0.001

* NSW excluded from analysis, as data were collected online only, as described in Materials and Methods; invalid/missing/other excluded, as only recorded for online (all paper surveys could be attributed to a specific state).

**Table 5:** Recruitment peaks for referrals from Facebook and mibss.org according to date, associated promotional activities, and respondent characteristics.

Peak	Dates	Promotional Activities	Number of Respondents Recruited during Peak (n)	Respondents’ Country of Birth (% of Peak Sample) ^1^		Respondents’ State of Residence (% of Peak Sample) ^1^	
Peak 1	23/09/2020–24/09/2020	Preceded by regular Facebook post captioned: “Together we can help improve community health” Coincided with advertisement in Queensland Chinese newspapers	12	China and Taiwan Other *p*-value	100.00% 0.00% <0.001	South Australia Not answered *p*-value	83.33% 16.67% 0.001
Peak 2	15/10/2020–16/10/2020	Preceded by regular Facebook post captioned: “If you’re interested in better sexual health for your community—we want to hear from you!” Coincided with post on Korean website Adelaide Focus (South Australia)	8	Korea Other *p*-value	75.00% 25.00% 0.282	South Australia Not answered *p*-value	87.50% 12.50% 0.015
Peak 3	07/11/2020–08/11/2020	Preceded by regular Facebook post captioned: “BBVs and STIs affect all groups” Coincided with posts on Korean websites MissyAUS (national) and Kangaroo (Victoria).	85	Korea Other *p*-value	90.59% 9.41% <0.001	Queensland South Australia Western Australia New South Wales Victoria Not answered *p*-value	22.35% 4.71% 7.06% 29.41% 23.53% 12.94% <0.001
Peak 4	11/11/2020–12/11/2020	Preceded by regular Facebook post captioned: “Together we can improve community health” Preceded by posts on Korean websites MissyAUS (national) and Kangaroo (Victoria).	8	Malaysia South Sudan Vietnam Hong Kong/Macau *p*-value	50.00% 12.50% 25.00% 12.50% 0.002	Western Australia New South Wales *p*-value	87.50% 12.50% <0.001
Peak 5	16/12/2020–17/12/2020	Preceded by regular Facebook post captioned: “Help us help your community” Coincided with paid Facebook advertising boost in Western Australia: “Calling all international students in WA on uni study break” Coincided with posts on Korean websites MissyAUS (national) and Kangaroo (Victoria). Coincided with recruitment at a public event/venue (Western Australia)	30	Indonesia Korea Somalia Hong Kong/Macau Not answered *p*-value	6.67% 66.67% 3.33% 6.67% 16.67% 0.510	Queensland Western Australia New South Wales Victoria Not answered *p*-value	16.67% 16.67% 26.67% 23.33% 16.67% 0.001
Peak 6	17/02/2021–18/02/2021	Preceded by regular Facebook post Coincided with post on Korean website MissyAUS (national)	10	Korea Other *p*-value	80.00% 20.00% 0.053	Queensland South Australia New South Wales Victoria Not answered *p*-value	10.00% 10.00% 40.00% 20.00% 20.00% 0.248

^1^*p*-values calculated by comparing responses collected during an individual peak (n) to other responses referred from Facebook and mibss.org (N-n, where N = 276).

**Table 6:** Outcomes of recruitment to paper surveys at public venues/events by date and venue.

State	Date	Event Description	Number of Recruitment Attempts	Number of Ineligible Persons Identified	Number of Surveys Refused	Reason for Refusal	Number of Respondents Recruited	Participation Rate ^ǂ^	Observations
Qld	28.11.20	World AIDS Day Event	55	3	3	Unknown	49	94.23%	N/A
	04.12.20	Inala Square Market (public space)	30	1	4	Too busy	8	27.59%	Those approached on way to work less likely to participate
					11	Not interested			
					5	Uncomfortable			
					1	Incapable			
	08.12.20	Inala Square Market	37	0	12	Too busy	6	16.22%	Men 30 years old seemed more likely to refuse the survey
					16	Not interested			
					3	Uncomfortable			
	02.12.20	STI and HPV Vaccination Workshop	13	0	0	N/A	13	100.00%	N/A
	16.3.21	Lunar New Year Lantern Festival (Sunshine Coast) *	12	0	2	Too busy	2	16.67%	Men are more likely to refuse. It is easier to recruit people among event staff/volunteers rather than participants/general public
WA	17.12.20	Christmas gathering	22	0	3	Too busy	5	22.73%	N/A
					2	Not interested			
					12	Took link for completion at later date			
	24.12.20	Christmas gathering	24	10	4	Too busy	5	35.71%	N/A
					1	Uncomfortable			
					1	Incapable			
					3	Took link for completion at later date			
	29.12.20	Wellington Square (public space)	19	3	2	Too busy	6	37.5%	N/A
					2	Not interested			
					3	Uncomfortable			
					3	Took link for completion at later date			
	13.01.21	Yanchep Observatory (tourist location)	50	0	50	Too busy	0	0.00%	N/A
	14.01.21	Fellowship Group	20	0	0	N/A	20	100.00%	N/A
	19.01.21	Church Fellowship Group	45	0	2	Too busy	5	11.11%	Women aged 65–75 and Indonesian people more likely to decline to participate
					1	Uncomfortable			
					37	Took link for completion at later date			
	21.01.21	Pinnacles (tourist location)	40	25	5	Too busy	5	33.33%	Women aged 50–70 more likely to decline to participate
					5	Uncomfortable			
	26.01.21	Yanchep Lavender Farm (tourist location)	51	7	10	Too busy	5	11.36%	N/A
					4	Not interested			
					3	Uncomfortable			
					2	Incapable			
					20	Took link for completion at later date			
	19.02.21	Hyde Park (public space)	18	5	1	Too busy	6	46.15%	N/A
					6	Took link for completion at later date			
	23.02.21	Fellowship group *	37	0	12	Took link for completion at later date	20	54.05%	N/A
TOTAL			473	54	251	N/A	155	36.99%	N/A

**^ǂ^** Denominator is number of people approached, excluding those known to be ineligible. * Potential error in recording data at this event, as number of recruitment attempts does not equal sum of number ineligible, refused, and completed.

**Table 7:** Participation rates for recruitment conducted among personal/organizational networks ^1^.

Type of Contact	Outcome	Number	Participation Rate ^2^
Face-to-face only (n = 211)	Ineligible	6	90.24%
	Survey completed	185	
	Took link for later completion	3	
	No response	2	
	Refused—too busy	4	
	Refused—uncomfortable	8	
	Refused—incapable of completing	3	
Telephone, email, or text message only (n = 88)	Ineligible	0	68.18%
	Survey completed	60	
	Took link for later completion	3	
	No response	3	
	Refused—too busy	6	
	Refused—not interested	7	
	Refused—uncomfortable	5	
	Refused—incapable of completing	2	
	Refused—no reason	2	
Mixture of face-to-face and telephone, email, or text message (n = 51)	Ineligible	2	77.55%
	Survey completed	38	
	Refused—too busy	4	
	Refused—not interested	1	
	Refused—uncomfortable	3	
	Refused—incapable	2	
	Refused—no reason	1	
TOTAL DOCUMENTED RECRUITMENT ATTEMPTS (n = 350)	Ineligible	8	82.75%
	Survey completed	283	
	Took link for later completion	6	
	No response	5	
	Refused—too busy	14	
	Refused—not interested	8	
	Refused—uncomfortable	16	
	Refused—incapable	7	
	Refused—no reason	3	

^1^ Excludes those who did not document number/type of contact attempts or outcome. ^2^ *p* = 0.001 (association between recruitment category and whether survey completed, excluding those known to be ineligible).

## Data Availability

Data supporting reported results can be obtained by contacting the corresponding author.

## Appendix A

**Use this form for any recruitment you try to do at public events or in a public location**

**RECORD OF RECRUITMENT EFFORTS**

Name of public event or description of public location: _____________________

Date: ______________________________________________________________

Name of peer researcher(s): _______________________________________________

	**Tally**
Number of **recruitment attempts** made ** This is the number of people you directly invited to complete the survey*	
Number of people who **completed** the survey	
Number of people **who took survey link** to complete at a later date	
Number of people **not eligible** to complete survey ** This is the number of people who you directly invited to complete the survey but were not eligible due to age, country of birth or length of time in Australia*	
Number of people who **refused to complete** the survey with reasons	
- No time/too busy	
- Not interested	
- Uncomfortable with subject matter	
- Incapable of completing survey (due to literacy, language or disability)	
- Other reason (describe)	
- Reason unknown	
Did you observe that any groups of people were **more likely to refuse** to complete the survey (e.g., men, women, people of a certain age, people from particular countries)?	
****We realise it is difficult to make these judgments so please answer as best you can*	
Is there anything else about your experiences recruiting at this event that you would like to share?	

**Please return this form to your line manager, together with your completed surveys, competition entry forms and iPad(s).**

## Appendix B

**Use this form for any people in your networks that you try to recruit**

**RECORD OF RECRUITMENT EFFORTS:**

Name of peer researcher: _______________________________________________

**Characteristics of Person (If Known)**	**How Contact Made—Attempt 1**	**How Contact Made—Attempt 2**	**How Contact Made—Attempt 3**	**Outcome** ***(Choose One Option)***
Gender:	□ In person □ Phone call □ Text message □ Email	□ In person □ Phone call □ Text message □ Email □ N/A	□ In person □ Phone call □ Text message □ Email □ N/A	□ Ineligible □ Survey completed □ Took survey link for completion at a later date □ No response □ Refused—too busy □ Refused—not interested □ Refused—uncomfortable □ Refused—incapable of completing (i.e., literacy, language) □ Refused—reason not given □ Refused—other
Actual or estimated age:				
Country of birth:				
Length of time in Australia:				
Gender:	□ In person □ Phone call □ Text message □ Email	□ In person □ Phone call □ Text message □ Email □ N/A	□ In person □ Phone call □ Text message □ Email □ N/A	□ Ineligible □ Survey completed □ Took survey link for completion at a later date □ No response □ Refused—too busy □ Refused—not interested □ Refused—uncomfortable □ Refused—incapable of completing (i.e., literacy, language) □ Refused—reason not given □ Refused—other
Actual or estimated age:				
Country of birth:				
Length of time in Australia:				

**Please return this form to your line manager, together with your completed surveys, competition entry forms and iPad(s).**
